# Is Traumatic Phlebitis a Contagious Disease?

**Published:** 1854-09

**Authors:** 


*Is Traumatic Phlebitis a contagious disease?*—A week or two ago, Dr. Simpson, of Edinburgh, was present at this Hospital on the operation day, and, among many matters which formed the subject of conversation on that occasion, the great prevalence of phlebitis after operations was one. Several suggestions were offered as possibly explanatory of this prevalence, and among them, the use of chloroform, the peculiar type of disease now existing, etc., all acknowledged to be very unsatisfactory. An opinion was broached by the Edinburgh Professor of much importance, and, if true, involving, we believe, a novel view of the pathology of the disease. Dr. Simpson stated his conviction, that phlebitis is essentially in the same category as puerperal fever, some forms of erysipelas, etc., and that it is capable of being conveyed from patient to patient by the Surgeon, just as the accoucheur is well known to convey the latter diseases. He believed, that the operator's hands, or even the knives used, might thus be made the means of transmitting the morbid poison. In support of the opinion, an instance was adduced in which a Surgeon had, on occount of the extreme prevalence of phlebitis among his patients, been obliged to desist for a time from operative practice. Mr. Stanley and Mr. Wormald, who took part in the conversation, both appeared to regard the suggestion as a valuable one, and not improbably true. We have mentioned the subject here, not with any intention hastily to either support or to invalidate the opinion, but because the question is of extreme importance, and it seemed well to attract the attention of our readers to it. Very possibly scattered fragments of evidence may be in existence, which, if collected and placed together, might go far to decide the question. As the subject is one which must engage our attention at a future time, we shall, meanwhile, be glad to have communicated to us any facts of importance in relation to it which may chance to be in possession of any of our readers.

Without in the least wishing to judge prematurely a theory propounded by so able a Physician, we can, however, scarcely help remarking, that there are certain circumstances connected with the history of the disease as it has shown itself in this Metropolis of late years, which militate strongly against the idea of its spreading by contagion. In the first place, its prevalence has been general, not limited to any particular Hospital, or to the practice of individual Surgeons. Secondly, although more frequent during some months than others, yet it has never markedly subsided, nor yet has it ever prevailed with an epidemic virulence in the least comparable to an outbreak of puerperal fever. When for a time more common in one Hospital, it has generally been about equally so in all others, as if dependent on general rather than on accidental causes. Thirdly, it has not, as far as we are aware, shown any tendency to prevail in particular wards, nor are we acquainted with any instance in which several patients occupying successively a bed on which a phlebitis patient has died, have suffered from the disease. (If there be any contagious principle, may it not be deemed far more probable, that the bed or the ward should be the means of communicating it, than the Surgeon's hands or instruments?) Fourthly, a not inconsiderable number of patients have suffered from the disease on whom no operation had been performed, and who were evidently affected with it at the time of admission into the Hospital. Among these, in the majority there has been the history of some slight wound or injury, but in a few the disease has appeared to be really idiopathic.

None of these objections are, of course, to be regarded as at all conclusive in themselves, but taken together they appear to throw a degree of doubt on the theory advanced by Dr. Simpson. It would be very important, if practicable, before commencing an examination into the peculiar features of the prevalent form of phlebitis, to ascertain whether its increasing frequency which is now so much talked about be real or only apparent. The symptoms of inflammation of veins, and its consequent pyæmia, are so much more generally known, and pathological examinations are so much more commonly undertaken now than formerly, that it is quite to be supposed that a considerable number of deaths now occurring should be correctly referred to that disease, which, in past years, might very likely have ranked under the heads of "hectic," "exhaustion," etc.—*Medical Times and Gazette, August,* 1854.

At a late meeting of the Royal Society of London, Dr. Theophilus Thompson read a paper, "On the changes produced in the Blood by the administration of Cod-liver oil and Cocoa-nut oil," of which the following is a brief extract:

The author has found that during the administration of cod-liver oil to phthisical patients their blood grew richer in red corpuscles, and he refers to a previous observation of Dr. Franz Simon to the same effect. The use of almond oil and of olive oil was not followed by any remedial effect, but from cocoa-nut oil results were obtained almost as decided as from the oil of the liver of the cod, and the author believes it may turn out to be a useful substitute. The oil employed was a pure cocoa oleine, obtained by pressure from crude cocoa-nut oil, as expressed in Ceylon and the Malabar coast from the *Copperah* or dried cocoa-nut kernel, and refined by being treated with an alkali and then repeatedly washed with distilled water. It burns with a faint blue flame, showing a comparatively small proportion of carbon, and is undrying.

The analysis of the blood was conducted by Mr. Dugald Campbell. The whole quantity abstracted having been weighed, the coagulum was drained on bibulous paper for four or five hours, weighed and divided into two portions. One portion was weighed and then dried in a water oven, to determine the water. The other was macerated in cold water until it became colorless, then moderately dried and digested with ether and alcohol to remove fat, and finally dried completely and weighed as fibrine. From the respective weights of the fibrine and the dry clot that of the corpuscles was calculated. The following were the results observed in seven different individuals affected with phthisis in different stages of advancement:

| | | Red corpuscles. | Fibrine. |
|---|---|---|---|
| First stage, before the use of cod-liver oil. | Female, | 129·26 | 4·52 |
| | Male, | 116·53 | 13·57 |
| First stage, after the use of cod-liver oil. | Female, | 136·47 | 5·00 |
| | Male, | 141·53 | 4·70 |
| Third stage, after the use of cod-liver oil.— | Male, | 138·74 | 2·23 |
| Third stage, after the use of cocoa-nut oil. | Male, | 139·95 | 2·31 |
| | Male, | 144·94 | 4·61 |

*Philosop. Mag. No.* 48.

